# Maternal BCG scar is associated with increased infant proinflammatory immune responses

**DOI:** 10.1016/j.vaccine.2016.11.079

**Published:** 2017-01-05

**Authors:** Patrice Akusa Mawa, Emily L. Webb, Abdelali Filali-Mouhim, Gyaviira Nkurunungi, Rafick-Pierre Sekaly, Swaib Abubaker Lule, Sarah Prentice, Stephen Nash, Hazel M. Dockrell, Alison M. Elliott, Stephen Cose

**Affiliations:** a MRC/UVRI Uganda Research Unit on AIDS, P.O. Box 49, Entebbe, Uganda; b London School of Hygiene & Tropical Medicine, Keppel Street, London WC1E 7HT, UK; c Case Western Reserve University School of Medicine, 10900 Euclid Ave., LC4960, Wood Bldg. W200, Cleveland, OH 44106, United States

**Keywords:** Maternal infections, Latent *Mycobacterium tuberculosis* infection, Maternal BCG scar, Infant innate responses, BCG immunisation, Tuberculosis, Heterologous effects

## Abstract

**Introduction:**

Prenatal exposures such as infections and immunisation may influence infant responses. We had an opportunity to undertake an analysis of innate responses in infants within the context of a study investigating the effects of maternal mycobacterial exposures and infection on BCG vaccine-induced responses in Ugandan infants.

**Material and methods:**

Maternal and cord blood samples from 29 mother-infant pairs were stimulated with innate stimuli for 24 h and cytokines and chemokines in supernatants were measured using the Luminex® assay. The associations between maternal latent *Mycobacterium tuberculosis* infection (LTBI), maternal BCG scar (adjusted for each other’s effect) and infant responses were examined using linear regression. Principal Component Analysis (PCA) was used to assess patterns of cytokine and chemokine responses. Gene expression profiles for pathways associated with maternal LTBI and with maternal BCG scar were examined using samples collected at one (n = 42) and six (n = 51) weeks after BCG immunisation using microarray.

**Results:**

Maternal LTBI was positively associated with infant IP-10 responses with an adjusted geometric mean ratio (aGMR) [95% confidence interval (CI)] of 5.10 [1.21, 21.48]. Maternal BCG scar showed strong and consistent associations with IFN-γ (aGMR 2.69 [1.15, 6.17]), IL-12p70 (1.95 [1.10, 3.55]), IL-10 (1.82 [1.07, 3.09]), VEGF (3.55 [1.07, 11.48]) and IP-10 (6.76 [1.17, 38.02]). Further assessment of the associations using PCA showed no differences for maternal LTBI, but maternal BCG scar was associated with higher scores for principal component (PC) 1 (median level of scores: 1.44 in scar-positive versus −0.94 in scar-negative, p = 0.020) in the infants. PC1 represented a controlled proinflammatory response. Interferon and inflammation response pathways were up-regulated in infants of mothers with LTBI at six weeks, and in infants of mothers with a BCG scar at one and six weeks after BCG immunisation.

**Conclusions:**

Maternal BCG scar had a stronger association with infant responses than maternal LTBI, with an increased proinflammatory immune profile.

## 1 Introduction

The bacillus Calmette-Guérin (BCG) vaccine protects against tuberculous meningitis and miliary tuberculosis (TB) in the infant [1], [2], [3], and also protects against leprosy [4]. However, the protective efficacy of BCG against pulmonary TB varies between populations, with latitude highlighted as an important factor for responses in adolescents and adults [1], [5], [6]. We recently investigated the effect of maternal latent *Mycobacterium tuberculosis* infection (LTBI) on the infant response to BCG immunisation [7], with results suggesting that maternal *M. tuberculosis* infection may impair adaptive immune responses in the infants, although a study in South Africa showed no such effect [8]. The associations with innate immune responses were not assessed.

Evidence that BCG immunisation may influence innate responses includes findings in both observational studies and randomized controlled trials that have highlighted the heterologous effects of BCG on childhood survival in both low- and high-income countries [9], [10], [11], [12], [13]. This has been suggested to be due to BCG-induced increases in function of the innate immune system, a phenomenon termed ‘trained immunity’ [14], [15], [16], [17], [18]. This is an observation of great global health significance, since mortality due to infectious agents other than TB is high in developing tropical countries [19].

One of the indicators of previous immunisation with BCG, in place of or in addition to vaccination records, is the presence or absence of a scar [20], [21], [22]. It has been shown that 52–97% of newborns administered BCG vaccine develop a scar, with differences depending on the strain of BCG vaccine used, the administrator and age of administration [20], [23], [24], [25], [26]. However, not all BCG vaccinated babies will scar. There are reports of a correlation between the presence of a scar and protection against TB [27], [28], as well as studies showing better survival with fewer respiratory infections [24], [29], [30], fewer skin infections and sepsis [31] in infants with a BCG scar.

Little is known about the link between the development of a BCG scar in mothers and immune responses in infants. We have previously observed that maternal BCG scar was associated with lower T helper (Th) 2 responses to crude culture filtrate proteins of mycobacteria in the infants [32]. In the context of a study designed to investigate the effects of maternal infections, including LTBI, on infant immune responses [7], we had the opportunity to also evaluate associations between maternal BCG scar and immune response profiles in the offspring.

## 2 Materials and methods

### 2.1 Study design, setting and ethical approval

The study design, settings, laboratory and clinical procedures have been described elsewhere [7]. Briefly, women residing within the study area and delivering in Entebbe General Hospital were eligible for inclusion. They were approached for consent, on admission in early labour, if they were willing to participate in the study, had a normal singleton pregnancy and were HIV negative. Cord blood was obtained at delivery, following consent. A questionnaire was completed to assess eligibility after delivery. The tuberculin skin test (TST, Statens Serum Institut, Copenhagen, Denmark) and T-SPOT.TB assay (Oxford Immunotec, Abingdon, UK) were used to test mothers for LTBI at approximately one week after delivery. Infants were then followed up to six weeks of life. This was an exploratory observational study in a relatively small number of subjects. The number of infants included in the study was chosen to be feasible within the time frame and resources available. The study was approved by the Uganda Virus Research Institute-Research and Ethics Committee, the Uganda National Council for Science and Technology and the London School of Hygiene & Tropical Medicine. Written, informed consent was obtained from participating women for themselves and their infant.

### 2.2 Immunological assays

Innate immune responses were measured in 29 mother-infant pairs using a whole blood assay (WBA) with supernatant analytes measured by Luminex®, and gene expression profiles were measured in infant samples obtained at one (n = 42) and six (n = 51) weeks after BCG immunisation using microarray.

### 2.3 Innate stimulation and measurement of responses using luminex® assay

Heparinized maternal and cord blood samples were diluted 1:1 with RPMI 1640 medium (Life Technologies Corporation, NY, USA) and stimulated with lipopolysaccharide (LPS) (toll-like receptor (TLR) 4 agonist, 100 ng/ml), FSL-1 (TLR2/6 agonist, 50 ng/ml), CpG-ODN2006 (TLR9 agonist, 5 μg/ml), CL097 (TLR7/8 agonist, 1 μg/ml) all from InvivoGen, San Diego, CA, USA, PAM3Cys-Ser (TLR1/2 agonist; ECM Microcollections GmbH, Tubingen, Germany; 100 ng/ml), Mannan (DC-SIGN agonist; Sigma-Aldrich; 100 μg/ml) and Curdlan (Dectin-1 agonist; Wako Chemicals GmbH, Neuss, Germany; 100 μg/ml). An unstimulated well was included to act as a negative control. After 24 h of incubation at 37 °C in 5% CO_2_, culture supernatants were harvested and stored at -80 °C for analysis of cytokines and chemokines. The concentrations of analytes in the culture supernatants were measured using a Bioplex multiplex cytokine assay system (Bio-Rad Laboratories, Hercules, CA, USA), following instructions from the manufacturer. A Bio-Plex 200 System (Bio-Rad Laboratories, Hercules, CA, USA) and the Bio-Plex Manager software (version 6.0; Bio-Rad Laboratories, Hercules, CA, USA) were used to run the samples. According to the manufacturer’s instructions, a curve fit was applied to standard curves, which were then used to extract sample concentrations. Limits of the assay working range (lower limit of quantification (LLOQ) and upper limit of quantification (ULOQ)) quoted by the manufacturer for each cytokine/chemokine were used to clean the data. For values below the acceptable range, half of the LLOQ was used and for values above the ULOQ, the ULOQ value for that particular analyte was used. The cytokines and chemokines analysed were IL-1β, IL-2, IL-4, IL-5, IL-6, IL-8, IL-10, IL-12p70, IL-13, IL-17A, IFN-γ, IP-10, MCP-1, MIP-1α, MIP-1β, RANTES, TNF-α, GM-CSF and VEGF.

### 2.4 RNA amplification and microarray

Gene expression microarrays were undertaken using unstimulated whole blood samples obtained from 42 and 51 infants at one and six weeks, respectively, to assess gene expression profiles after BCG immunisation. The Illumina RNA Amplification Kit (Ambion, Austin, TX, USA) was used to amplify a median of 124 ng (range 63–174 ng) of the extracted RNA. A Biotin-16-UTP label was incorporated into amplified RNA during the *in vitro* transcription process (Perkin Elmer Life and Analytical Sciences, Woodbridge, Ontario, Canada). Amplification gave yields ranging from 1 μg to 25 μg. Amplified RNA (1000 ng per array) was hybridized to the IlluminaHumanHT-12_V4 BeadChip according to the manufacturer’s instructions (Illumina, San Diego, CA, USA). The IlluminaHumanHT-12_V4 bead chip comprises 42,000 sequences representing 31,000 annotated genes from the curated portion of the NIH Reference Sequence Database (http://www.ncbi.nlm.nih.gov/RefSeq/). Each sequence is represented at least 30 times on the array. Arrays were scanned with an Illumina bead array confocal scanner, according to the manufacturer’s instructions. Array data processing and analysis was performed using Illumina BeadStudio software.

### 2.5 Statistical analysis

The objective of this analysis was to investigate the effects of maternal latent TB and helminth infection on infant innate immune responses. In the event, helminth infections were rare in this study group [7], so the principal exposures considered were maternal LTBI and maternal BCG scar. In the multivariate analysis, the effects of maternal LTBI and maternal BCG scar were adjusted for. Maternal and infant factors such as maternal age, gravidity status, infant birth weight and gender were not crudely associated with infant responses and were not adjusted for, and the numbers involved were generally small.

Cytokine and chemokine concentrations showed skewed distributions. Results were transformed to log_10_ (cytokine concentration + 1) for graphical representation using GraphPad Prism v6.0c (GraphPad software, Inc., La Jolla, CA, USA) and for analysis by linear regression using bootstrapping [33] using STATA v. 13.1 (College Station, TX, USA). Results from regression analyses are presented as adjusted geometric mean ratios (aGMR) [95% confidence interval (CI)]. Multiplex data values below the lowest concentration were assigned as 1.6 pg/ml. Unstimulated responses were subtracted from antigen-stimulated results and negative values were set to zero. The Mann–Whitney *U* test was used to compare responses between infants of mothers with and without LTBI and those with and without a BCG scar and correlation between two continuous variables was assessed using the spearman rho test. For the different stimuli, the median maternal and cord blood responses, as well as the associations of infant responses with maternal LTBI and maternal BCG scar were analysed. In addition to looking at single cytokines and chemokines, Principal Component Analysis (PCA) [34] was performed on the cytokine and chemokine variables to summarize them. For this, an average cytokine or chemokine response was worked out for each infant by calculating the mean concentration obtained from the seven different stimuli (after subtracting unstimulated responses). The R programme (v3.2.2. R Foundation for Statistical Computing, Vienna, Austria) was used for further assessment of the associations.

For microarray, raw Illumina probe data were exported from BeadStudio and screened for quality. Pre-processing and statistical analysis was conducted using the R statistical language and various software packages from Bioconductor [35]. Quantile normalization was applied, followed by a log_2_ transformation. The LIMMA package was used to fit a linear model to each probe and (moderated) *t* tests or *F* tests were performed on the groups being compared. To control the expected proportions of false positives, the FDR for each unadjusted *p* value was calculated using the Benjamini and Hockberg method implemented in LIMMA. The microarray data are available through the National Center for Biotechnology Information Gene Expression Omnibus (GSE87801). Pathway analysis was performed using Gene Set Enrichment Analysis (GSEA), a non-parametric annotation-driven statistical analysis method [36], to assess which biological processes are associated with the different LTBI and BCG scar groups. We tested gene sets from the Molecular signature Database (MsigDB, http://www.broad.mit.edu/gsea/msigdb Hallmark collection (h.all.v5.0.symbols.gmt) which summarize and represent specific well-defined biological states or processes displaying coherent expression. Statistical significance was set for *p* value below 0.05.

## 3 Results

### 3.1 Participant characteristics

The flow of the participants through the study and recruitment details have been described elsewhere [7]. Of the twenty-nine mothers considered for the WBA/Luminex analysis, 12 had a LTBI and 16 had a BCG scar. Three mothers had missing information on BCG scar and were not included in the analysis. Mothers with and without a BCG scar were comparable in terms of age (25 years versus 26 years, p = 0.78), LTBI (31% versus 50%, p = 0.42) and gravidity status (37% versus 50% primigravida, p = 0.70). Their infants were comparable in terms of birth weight (3.09 versus 3.22, p = 0.47) and gender (19% versus 40% male, p = 0.38). Ninety-three mothers were considered for the gene expression microarray, and of these, 21 had a LTBI and 38 had a BCG scar. Mothers with and without a BCG scar were comparable in terms of age (24 years versus 25 years, p = 0.34), LTBI (26% versus 41%, p = 0.26), gravidity status (39% versus 45% primigravida, p = 0.78). Their infants were comparable in terms of birth weight (3.24 versus 3.21, p = 0.77) and gender (40% versus 47% male, p = 0.77) (Table 1).

**Table 1:** Characteristics of participants by maternal BCG scar status. The figures are given as numbers with percentage (%) in brackets, or as mean values. *P* value is based on unmatched *t* test for differences in maternal age and infant birth weight, and a two-sided Fisher’s exact test for differences in maternal LTBI, parity and infant gender between scar-positive and scar-negative groups.

Characteristics	Participants for Luminex assay			Participants for microarray		
	Maternal BCG Scar present (n = 16)	Maternal BCG Scar absent (n = 10)	*P* value	Maternal BCG Scar present (n = 38)	MaternalBCG Scar absent (n = 22)	*P* value
*Mothers*						
Age, mean (years)	25	26	0.78	24	25	0.39
Latent TBI status, Present, no (%)	5 (31)	5 (50)	0.42	10 (26)	9 (41)	0.26
Gravidity, Primigravida, no (%)	6 (37)	5 (50)	0.70	14 (39)	10 (45)	0.78
*Infants*						
Sex, Male, no (%)	3 (19)	4 (40)	0.38	14 (40)	8 (47)	0.77
Mean birth weight (kg)	3.09	3.22	0.47	3.24	3.21	0.77

### 3.2 The innate immune responses to the different stimuli

The median cytokine and chemokine responses to the different stimuli were analysed. Supplementary Tables 1A and 1B illustrate these for mothers and infants, respectively. There were overall low to moderate concentrations of cytokines, chemokines and growth factors in both maternal and cord blood samples, except for IL-6, IL-8, MCP-1, MIP-1α, MIP-1β and IP-10 (to TLR 7/8 agonist) where concentrations were high across the different stimuli.

### 3.3 The association between maternal LTBI, maternal BCG scar and innate immune responses in mothers and their offspring

Cytokine and chemokine responses were analysed for associations with maternal LTBI and maternal BCG scar.

**Fig. 1:**
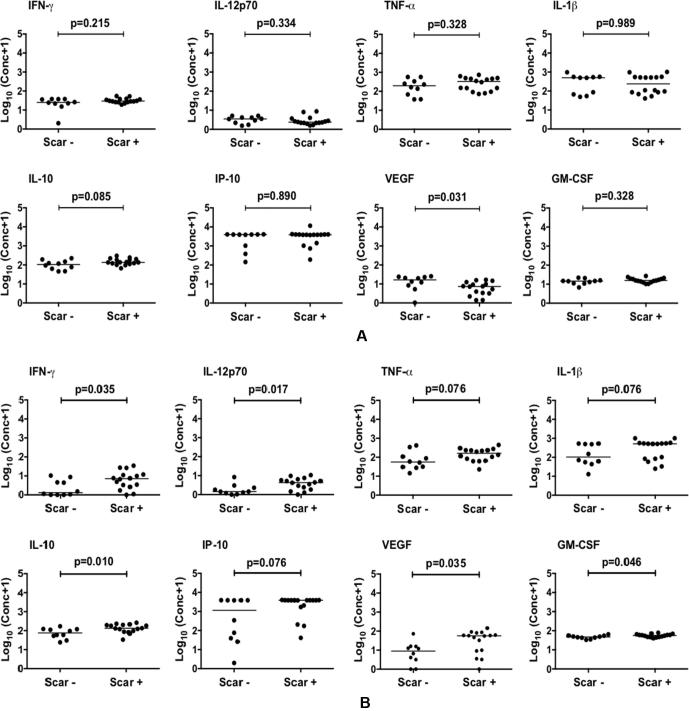
The association between maternal BCG scar and infant innate responses. Combined median cytokine or chemokine production following overnight stimulation with lipopolysaccharide (LPS) (toll-like receptor (TLR) 4 agonist), FSL-1 (TLR2/6 agonist), CpG-ODN2006 (TLR9 agonist), PAM3Cys-Ser (TLR1/2 agonist), CL097 (TLR7/8 agonist), Mannan (DC-SIGN agonist) and Curdlan (Dectin-1 agonist). Cytokines representing Th1/proinflammatory (IFN-γ, IL-12p70, TNF-α and IL-1β), immunoregulatory responses (IL-10) and chemokines/growth factors (IP-10, VEGF and GM-CSF) measured by Luminex® assay are shown for the mothers’ blood (Fig. 1A) and for infants’ cord blood (Fig. 1B). Data presentation was performed using GraphPad Prism.

For the combined results, maternal responses were not associated with their own BCG scar, except for VEGF where mothers without a BCG scar, compared to those with, had higher concentrations (p = 0.031, Fig. 1A). For IL-4, mothers with a BCG scar, compared to those without, had higher responses (p = 0.012, Supplementary Table 1). Maternal LTBI was positively associated with cord blood IP-10 responses, with an aGMR [95% CI] of 5.10 [1.21, 21.48], p = 0.026 (data not shown).

Cord blood samples obtained from infants of mothers with a BCG scar, compared to those without BCG scar, had overall higher responses to innate stimuli for the following analytes: IFN-γ (aGMR 2.69 [1.15, 6.17]), IL-12p70 (1.95 [1.10, 3.55]), IL-10 (1.82 [1.07, 3.09]), VEGF (3.55 [1.07, 11.48]) and IP-10 (6.76 [1.17, 38.02] There was a similar, but weaker, trend for the proinflammatory cytokines TNF-α (aGMR 1.99 [0.69, 5.89]) and IL-1β (1.55 [0.37, 6.61]). (Fig. 1B, and Supplementary Tables 2 and 3).

The associations between infant responses to the different stimuli and maternal LTBI (Supplementary Figs. 1A and 1B) and maternal BCG scar (Supplementary Figs. 2A and 2B) were analysed. The following CpG-specific cytokine and chemokines were positively associated with maternal LTBI: IL-12p70 (p = 0.014), MCP-1 (p = 0.011) and MIP-1β (p = 0.007) (Supplementary Fig. 1B). Cytokines and chemokines that were positively associated with maternal BCG scar included: IL-10 (p = 0.017) and GM-CSF (p = 0.042) to PAM3Cys-Ser; TNF-α (p = 0.044), IL-2 (p = 0.019), IL-1β (0.005), IL-6 (p = 0.017), IL-10 (p = 0.001), GM-CSF (p = 0.014) and VEGF (p = 0.048) to FSL-1; TNF-α (0.017) to LPS; IFN-γ (p = 0.018), IL-12p70 (p = 0.023), GM-CSF (p = 0.047) to CL097; IL-2 (p = 0.048), IL-1β (0.017), IL-10 (p = 0.040), IL-8 (p = 0.011), GM-CSF (p = 0.027) to Mannan; TNF-α (p = 0.027), IL-12p70 (P = 0.012) and VEGF (P = 0.003) to Curdlan (Supplementary Figs. 2A and 2B).

### 3.4 Principle component analysis of infant innate immune responses

**Fig. 2:**
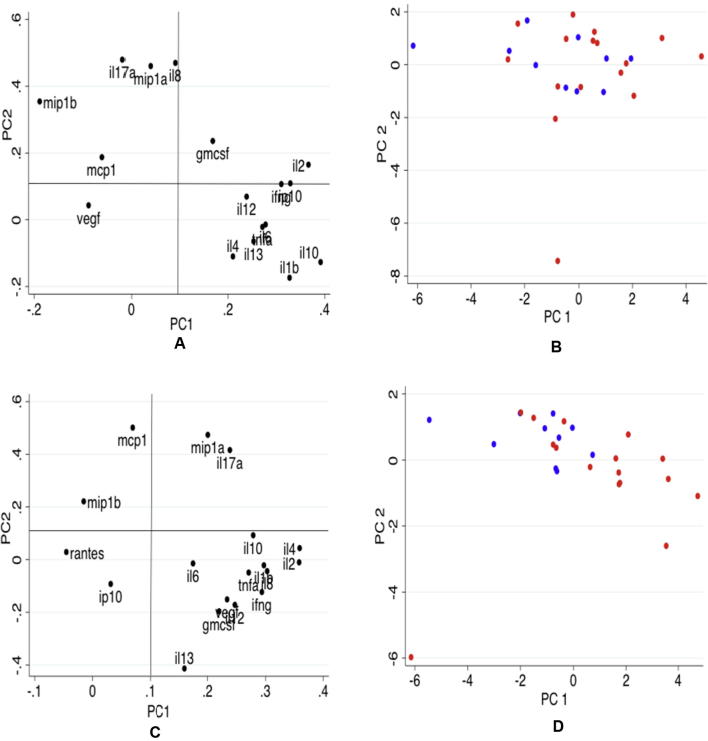
Scatterplots of first and second factor loadings for maternal and cord blood, derived from Principal Component Analysis of 17 analytes, showing cytokines and chemokines (A and C), individual mothers (B) and neonates (D). For mothers, the first principal component (PC) was characterized by a mixture of cytokines and the second PC consisted of chemokines. For neonates, the first PC was characterized by proinflammatory cytokines and the second PC consisted of chemokines, based on factor loadings >0.1. Red circles represent BCG scar-positive (Scar+) mothers and their infants. BCG scar-negative (Scar−) mothers and their infants are represented by blue triangles. One infant had overall high background responses (unstimulated samples) for most cytokines/chemokines measured. Subtracting the unstimulated values from antigen stimulated values gave overall low net values, thus the negative PC scores (−6.311 for PC1 and −6.228 for PC2). (For interpretation of the references to colour in this figure legend, the reader is referred to the web version of this article.)

We observed correlations among the cytokines and chemokines measured and this was summarized using PCA. For the mothers, two principle components (PCs) were identified, which together, accounted for 43% of the variance in the dataset. The first PC explained 25% of the total variance and was characterized by IFN-γ, TNF-α, IL-12p70, IL-1β, IL-6, IL-4, IL-10, IL-13 and the second PC explained a further 18% of the total variance and was characterized by MCP-1, MIP-1α, MIP-1β, IL-8, and IL-17A based on factor loadings > 0.1 (Fig. 2A). Neither Maternal LTBI (data not shown) nor maternal BCG scar (Fig. 2B) was associated with the mothers’ own PC scores.

For the infants, two PCs identified accounted for 53% of the variance in the dataset. The first PC explained 39% of the total variance and was characterized by most of the cytokines and growth factors measured (IFN-γ, TNF-α, IL2, IL-12p70, IL-4, IL-13, IL-10, IL-1β, IL-6, IL-8, VEGF and GM-CSF) (Fig. 2C). The second PC explained a further 14% of the total variance and was characterized by MCP-1 and MIP-1β. Infants with a high response in PC1 were born to mothers with a BCG scar (Fig. 2D).

**Fig. 3:**
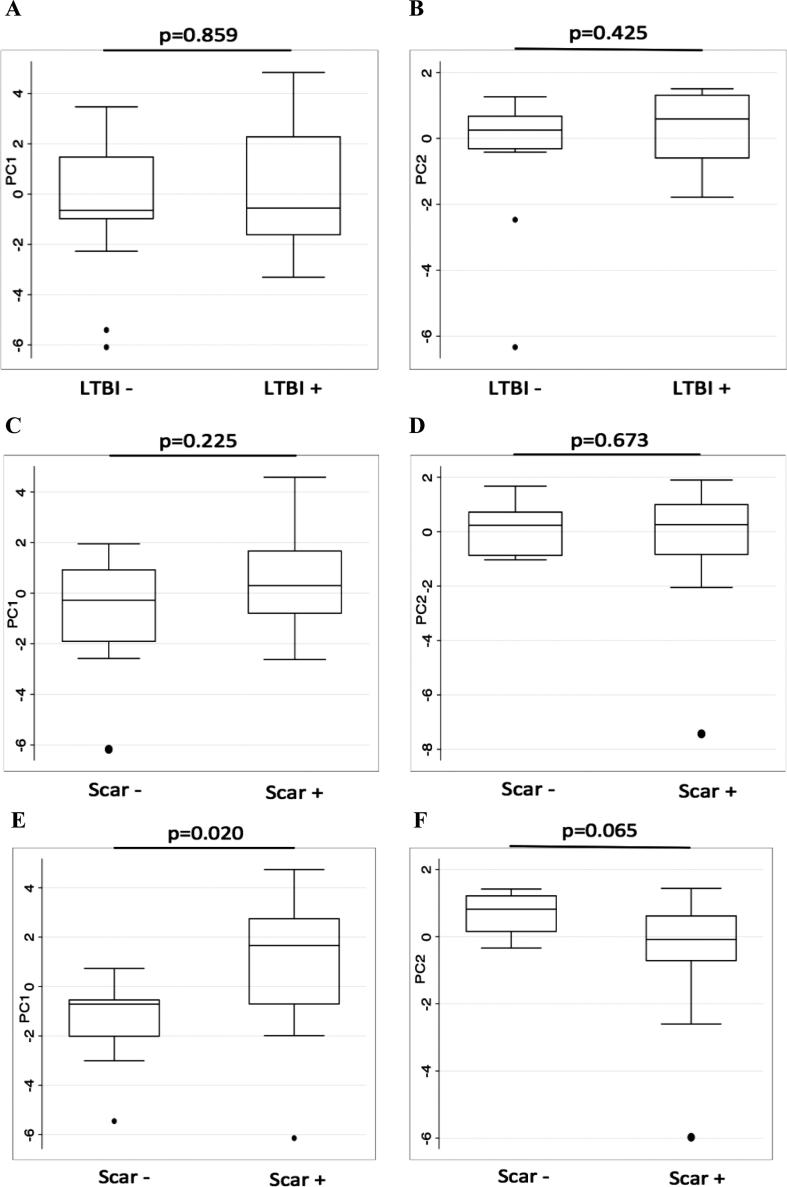
The association between maternal LTBI, maternal BCG scar and the innate immune responses in mothers and neonates. PCA was used to assess the association between maternal LTBI, maternal BCG scar and infant responses. The association between maternal LTBI and infant innate responses (A and B), and the association between maternal BCG scar and maternal (C and D) and infant (E and F) responses are shown. Two PCs that explained 43% and 53% of the variance in the dataset for mothers and neonates, respectively, were identified. The box plots represent the median and the interquartile range of the levels of the two PCs. The whiskers show the minimum and maximum values. *P* values are from Wilcoxon rank sum test.

These results are illustrated in Fig. 3. There were no associations between maternal LTBI and levels of PCs in the infants (Fig. 3A and B), and no associations between maternal BCG scar and levels of PCs in the mothers (Fig. 3C and D). Maternal BCG scar was associated with high levels of PC1 in the infants (median level of scores: 1.44 in scar-positive versus −0.94 in scar-negative, p = 0.020, Fig. 3E). There was no association between maternal BCG scar and levels of PC2 in the infants (median level of scores: −0.002 in scar-positive versus 0.754 in scar-negative, p = 0.065, Fig. 3F).

The correlations among the cytokines and chemokines measured are shown in Supplementary Table 4.

### 3.5 Analyses of clusters of innate cytokines and chemokines

**Fig. 4:**
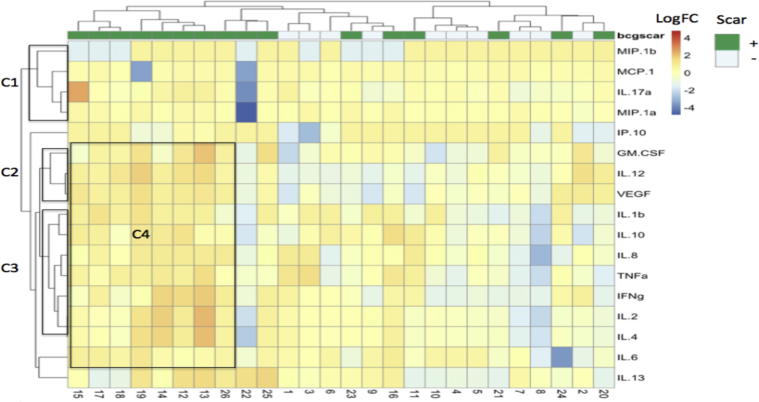
Cluster analysis of the innate cytokines and chemokines using the average linkage distance between clusters using R. Clusters go from root to leaf node for each cytokine and for the individual infants. Clusters in between are based on their agglomerative value. The branch shows the similarity, the shorter the branch, the more similar. Expression levels of individual cytokines (log_10_ [pg/ml]) are represented by shades of blue to red based on their correlations according to the dendrogram on the left, with highest values in dark red and the lowest in dark blue. Three distinct sets of correlated cytokines “clusters” are indicated as C1, C2 and C3 on the left. In addition, eleven cytokines (C4) form a cluster that has mainly inflammatory cytokines. Most infants of mothers with a BCG scar (top, green) clustered together in one discrete group, distinct from infants of mothers without a BCG scar (top, light blue). (For interpretation of the references to colour in this figure legend, the reader is referred to the web version of this article.)

In addition to the PCA, we performed a hierarchical bicluster analysis of the innate responses to further identify sets of cytokines and chemokines that might be coordinately expressed in infants of mothers with and without a BCG scar using R programming. Three clusters (C) of cytokines were identified (illustrated in Fig. 4): MCP-1, MIP-1α, MIP-1β, IL-17A (C1), VEGF, GM-CSF, IL-12p70 (C2) and IL-1β, IL-8, TNFα, IFN-γ, IL-2, IL-4, IL-10 (C3). Eleven cytokines formed an additional cluster (C4) that contained high concentrations of the proinflammatory cytokines produced by infants of mothers with a BCG scar.

### 3.6 Gene expression profiles in infants of mothers with and without LTBI, and in the infants of mothers with and without a BCG scar

**Fig. 5:**
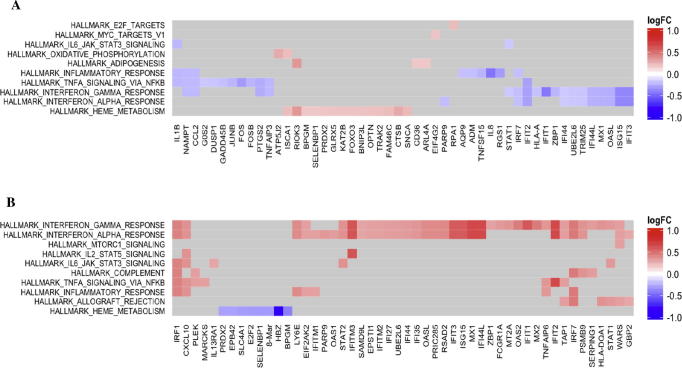
Gene Set Enrichment Analysis for the comparison of infants of mothers with and without LTBI. A checkerboard map showing top enriched pathways on y-axis and top leading edge genes (gene members contributing most to the enrichment score) on the x-axis. Scale at the right represents the gene expression fold change (log2 (exposed/unexposed)). Red (blue) indicates genes that are up-regulated (down-regulated) among infants of mothers with LTBI mothers. Interferon and inflammation response pathways were up regulated in infants of mothers with LTBI at six weeks. FDR adjusted *p*-value cut off of <0.25 was applied for pathways significance. (For interpretation of the references to colour in this figure legend, the reader is referred to the web version of this article.)

**Fig. 6:**
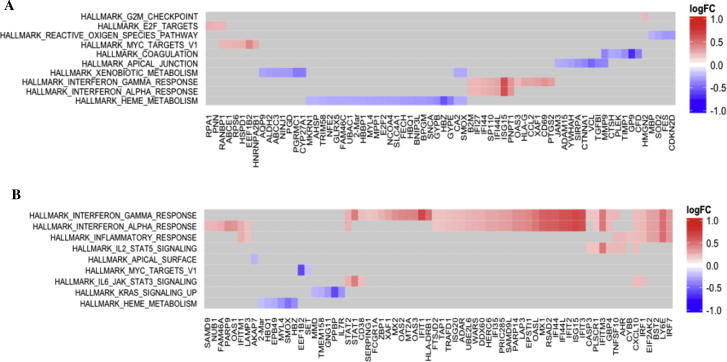
Gene Set Enrichment Analysis for the comparison of infants of mothers with and without a BCG scar. A checkerboard map is presented showing top enriched pathways on y-axis and top leading edge genes (gene members contributing most to the enrichment score) on the x-axis. Scale at the right represents the gene expression fold change (log2 (scar+/scar−). Red (blue) indicates genes that are up-regulated (down-regulated) among infants of scar-positive mothers. Interferon and inflammation response pathways are up regulated in infants of mothers with a BCG scar at one and six weeks after BCG immunisation. FDR adjusted p-value cut off of <0.25 was applied for pathways significance. (For interpretation of the references to colour in this figure legend, the reader is referred to the web version of this article.)

In order to further examine the associations we found with the innate responses using the Luminex® assay, gene expression microarray analysis was performed using blood obtained from 42 and 51 infants at one and six weeks post-BCG, respectively, using RNA extracted from unstimulated whole blood. Gene expression from infants of mothers with and without LTBI and those with and without a BCG scar were compared. Infants of mothers with LTBI, compared to those of mothers without LTBI, had down-regulated interferon and inflammation pathways one week after BCG immunisation (Fig. 5A), but up-regulated interferon and inflammation pathways at six weeks post immunisation (Fig. 5B). In contrast, the interferon and inflammation pathways were both up regulated in infants of mothers with a BCG scar at one (Fig. 6A and Supplementary Fig. 3A) and six (Fig. 6B and Supplementary Fig. 3B) weeks after BCG immunisation.

## 4 Discussion

This study reports an unexpected finding about the association between maternal BCG scar and infant responses in a birth cohort. We have shown that infants of mothers with a BCG scar have enhanced proinflammatory responses. The concentrations of proinflammatory cytokines measured in cord blood in response to stimulation with innate stimuli using the Luminex® assay were increased in infants of mothers with a BCG scar. The expression of genes in the interferon and inflammation responses pathways measured using gene transcription microarray was also increased in infants of mothers with LTBI at six week post BCG immunisation, and in infants of mothers with a BCG scar at one and six weeks after BCG immunisation.

Innate immune responses may determine the effectiveness of adaptive responses [37] and lead to either biased [38] or regulatory immune profiles [39], [40], [41]. The increased responses reported here may therefore impact on immune responses to vaccines administered at birth and on the course of infections and disease in childhood. Further studies of human innate immune profiles in response to immunisation, and during infections and disease, are needed.

There were no associations between maternal BCG scar and the mothers’ own innate immune responses: associations were manifested only in the infants. The presence of a maternal BCG scar was taken to indicate BCG immunisation of a mother during infancy. There are suggestions of positive associations between IFN-γ responses and reactions at the site of BCG immunisation [42], [43], and presence of a scar has been associated (in other studies) with protection against LTBI [27], [28]. Scar might therefore be a good measure of protective immune responses. However, it is difficult to reconcile how a response to a vaccine administered to mothers in their infancy would exert its effects several years later in the offspring. It is possible that there may be common genetic factors between the mothers and their infants that determine scar formation and subsequent responses in the infants, or that the factors associated with scar formation in the mothers are transmitted to the infants. The lack of association between maternal BCG scar and the mother’s own responses could be attributed to cumulative life-time exposures that alter the initial maternal innate immune responses after BCG immunisation. We did not collect data on scarring in these infants, but an ongoing larger study with a longer follow up will provide the opportunity to assess relationships between scarring and immune responses in mothers and their infants.

The development of a scar is also dependent upon the strain, dose and method of administration of the BCG vaccine [44]. The Danish strain of BCG vaccine, compared to BCG Russia, has been shown to elicit stronger responses in infants one year later and to cause more scarring [23], [24], [25], [45], [46], and the intradermal route of administration is associated with the formation of distinctive scars [47], [48]. We were unable to ascertain the strain, the dose and the method of administration of BCG vaccine in these women, although the most common strain and the method used in this setting are BCG Russia and the intradermal method, respectively. Since BCG immunisation is administered in the neonatal period, it is difficult to obtain information about BCG immunisation status of adults in a country where hospitals do not routinely record vaccine strain. There is therefore the possibility of misclassification of women based on the presence or absence of a scar. It is possible that the scar-negative women may have been BCG vaccinated without developing a scar, or that scars were lost with time. Our observed differences in infant response may therefore relate either to the mother’s BCG immunisation status or to the quality of the mother’s response to BCG immunisation.

Previous studies have reported the presence [49], [50] or absence [51] of maternal cells in cord blood samples. It is therefore possible that the high proinflammatory response observed in cord blood could be due to responses from maternal cells in cord blood, but the method we used for collecting cord blood (by needle and syringe, with no “milking” of the cord, coupled with the use of trained midwives) minimized contamination. Previous tests carried out on maternal and cord blood samples in our studies (comparing levels of β-human chorionic gonadotropin) showed that contamination of cord blood by maternal blood was rare (unpublished data).

Interferon and inflammatory pathways were down-regulated in infants of mothers with LTBI at one week, but up-regulated at six weeks after BCG immunisation; this offers some support to the hypothesis that prenatal exposure to maternal LTBI modifies the infant response to BCG, but the change in direction of effect as the immune response matured was unexpected, and these findings would need to be confirmed in a larger study.

Limitations of the study were its observational and explorative nature, its small sample size relative to the many outcomes assessed. Maternal and infant factors such as maternal age, gravidity status, infant birth weight and gender were not adjusted for since these were not crudely associated with infant responses, and the numbers involved were generally small.

In summary, maternal BCG scar had a stronger association with infant responses than maternal LTBI, with an increased proinflammatory profile of immune responses. The mechanisms that underlie this association need to be further examined in a larger study.

## Funding statement

This work was supported by the European Community’s Seventh Framework Programme (FP7/2007-2013) under EC-GA no. 241642. PAM was also supported by a Commonwealth PhD Fellowship, AME by a Welcome Trust Senior Fellowship (grant number 095778), SC by 10.13039/100004440Wellcome Trust funding (grant number 084344) and PAM and SC by an MRC project grant (MR/K019708).

## Author contributions

A.M.E. conceived the study. P.A.M., S.C. and A.M.E. designed the study. P.A.M. coordinated the study (together with S.A.L.), carried out the immunoassays under the supervision of S.C., performed the data analysis under the supervision of E.L.W. and S.N, drafted the manuscript and coordinated the writing of the manuscript. A.F. conducted the microarray assays. G.N. performed the T-SPOT.TB assays. P.A.M., E.L.W., H.M.D., A.M.E., S.C., A.F., G.N., R.P.S., S.P., S.N., and S.C. contributed to discussion and interpretation of the results. P.A.M., A.F., H.M.D., A.M.E. and S.C. participated in writing the manuscript. All the authors read and approved the final manuscript.

## Conflict of interest statement

The authors have no associations that might pose a conflict of interest.

## Appendix A Supplementary material

Supplementary Table 1 Cytokine and chemokine responses to individual innate stimuli, showing responses for the mothers (A) and the infants (B). The values are median responses in pg/ml.

Supplementary Table 2 Cytokine and chemokine responses in mothers with and without a BCG scar, and in their infants, measured by Luminex® assay. The values are shown as medians in pg/ml with the interquartile range in brackets.

Supplementary Table 3 The association between maternal BCG scar and infant innate responses. The values are shown as crude and adjusted geometric means ratios (GMR) with 95% confidence interval (CI).

Supplementary Table 4 Pairwise spearman rank correlation coefficients for cytokines and chemokines. Correlation between the cytokines and chemokines was assessed using the spearman rho test. *P* values less than 0.05 are shown in bold with † sign.

Supplementary Fig. 1 Cytokine and chemokine responses to innate stimuli, showing responses in maternal blood post-delivery (A) and cord blood (B). Clear and grey bars represent infants of mothers without and with LTBI, respectively. The horizontal lines represent the median. Statistically significant differences are shown by asterisk (∗).

Supplementary Fig. 2 Cytokine and chemokine responses to innate stimuli, showing responses in maternal blood post-delivery (A) and cord blood (B). Clear and grey bars represent infants of mothers without and with a BCG scar, respectively. The horizontal lines represent the median. Statistically significant differences are shown by asterisk (∗).

Supplementary Fig. 3 Heatmap showing the level of expression of the differentially expressed genes between infants of mothers with and without a BCG scar measured at one (A) and six (B) weeks post-BCG immunisation. The genes selected are the top significantly differentially expressed genes selected on fold change (1.3 fold up- or down- regulation) and *p* value (p < 0.05) basis. The colour scale shows the level of gene expression (scaled across infants) using *z* score, where red and green correspond to up- and down-regulation, respectively.
