# Supplementary material for: Maternal BCG scar is associated with increased infant proinflammatory immune responses
Source: Vaccine. 2017 Jan 5;35(2):273–82. doi: 10.1016/j.vaccine.2016.11.079 (PMC5357573; doi:10.1016/j.vaccine.2016.11.079)
Supplement: Supplementary Table 1 — Cytokine and chemokine responses to individual innate stimuli, showing responses for the mothers (A) and the infants (B). The values are median responses in pg/ml. [file mmc1.docx]

**Supplementary Table 1**

**(A)**

| Cytokine/  chemokine | PAM3Cys-Ser (TLR 1/2 agonist) | FSL-1  (TLR 2/6 agonist) | LPS  (TLR 4 agonist) | CL097  (TLR 7/8 agonist) | CpGODN2006  (TLR 9 agonist) | Mannan  (DC-SIGN agonist) | CURD  (DECTIN-1 agonist) |
| --- | --- | --- | --- | --- | --- | --- | --- |
| IFN-γ | 33.29 | 15.22 | 43.17 | 42.75 | 1.1399 | 10.31 | 49.81 |
| TNF-α | 51.22 | 5.15 | 350.52 | 262.39 | 0 | 3.24 | 854.78 |
| IL-2 | 2.86 | 0.95 | 4.04 | 3.07 | 0 | 0.75 | 4.21 |
| IL-12p70 | 0.45 | 0 | 2.27 | 2.98 | 0 | 0.929 | 2.61 |
| IL-1β | 17.32 | 0.82 | 149.47 | 121.94 | 0 | 0 | 3255.72 |
| IL-6 | 188342.53 | 183.94 | 18871.69 | 18861.84 | 0.67 | 18.43 | 18875.71 |
| IL-4 | 0.08 | 0 | 0.26 | 0 | 0 | 0 | 0.33 |
| IL-5 | 1.55 | 1.55 | 1.55 | 1.55 | 1.55 | 1.55 | 1.55 |
| IL-13 | 0.49 | 0 | 0.69 | 0 | 0 | 0 | 4.09 |
| IL-10 | 75.53 | 16.22 | 206.31 | 156.46 | 0 | 1.44 | 454.08 |
| IL-17A | 21.76 | 15.03 | 26.85 | 23.84 | 1 | 9.98 | 31.54 |
| IP-10 | 5.50 | 39.13 | 231.29 | 26710.98 | 0 | 26.39 | 378.16 |
| IL-8 | 26403 | 981.56 | 26403 | 890.59 | 203.7 | 1389.14 | 26403 |
| GM-CSF | 33.34 | 23.9 | 45.34 | 35.74 | 18.17 | 22.61 | 43.9 |
| VEGF | 0 | 0 | 9.84 | 1.09 | 1.80 | 3.92 | 21.75 |
| MCP-1 | 1445.21 | 1445.21 | 1445.15 | 1339.21 | 0 | 1401.2 | 1059.21 |
| MIP-1α | 831.82 | 47.91 | 831.83 | 831.82 | 1.78 | 25.84 | 831.83 |
| MIP-1β | 1533.27 | 1533.27 | 1533.27 | 1529.29 | 0 | 100 | 1533.27 |
| RANTES | 0 | 0 | 0 | 0 | 0 | 0 | 0 |

**(B)**

| Cytokine/  chemokine | PAM3Cys-Ser (TLR 1/2 agonist) | FSL-1  (TLR 2/6 agonist) | LPS  (TLR 4 agonist) | CL097  (TLR 7/8 agonist) | CpGODN2006  (TLR 9 agonist) | Mannan  (DC-SIGN agonist) | CURD  (DECTIN-1 agonist) |
| --- | --- | --- | --- | --- | --- | --- | --- |
| IFN-γ | 0 | 0 | 5 | 5 | 0 | 0 | 10 |
| TNF-α | 51.76 | 7.05 | 245.18 | 142.02 | 0 | 3.61 | 396.22 |
| IL-2 | 2 | 1 | 3 | 3 | 0 | 0 | 3 |
| IL-12p70 | 2 | 1 | 2 | 0 | 0 | 1 | 4 |
| IL-1β | 31 | 4 | 130 | 125 | 0 | 2 | 3257 |
| IL-6 | 16025 | 338 | 18873 | 18765 | 0 | 44 | 18873 |
| IL-4 | 0 | 0 | 0 | 0 | 0 | 0 | 0 |
| IL-5 | 1 | 1 | 1 | 1 | 1 | 1 | 1 |
| IL-13 | 0.63 | 0.23 | 0 | 2 | 0 | 0 | 4 |
| IL-10 | 67 | 11 | 108 | 156 | 0 | 2 | 483 |
| IL-17A | 27 | 20 | 31 | 29 | 2 | 15 | 33 |
| IP-10 | 5 | 9 | 15 | 26756 | 0 | 4 | 14 |
| IL-8 | 26403 | 1532.49 | 26403 | 26403 | 181.31 | 2712.5 | 26403 |
| GM-CSF | 55 | 45 | 62.36 | 55 | 39 | 42 | 64 |
| VEGF | 37.17 | 9.69 | 6.60 | 0.91 | 0.23 | 13.41 | 62.42 |
| MCP-1 | 1797 | 1597 | 1794 | 1797 | 0 | 1597 | 1798 |
| MIP-1α | 831 | 73 | 832 | 832 | 0 | 18 | 832 |
| MIP-1β | 1545 | 1544.98 | 1545 | 1544.98 | 0 | 1516.97 | 1544.98 |
| RANTES | 0 | 0 | 0 | 0 | 0 | 0 | 0 |
