# Supplementary material for: Maternal BCG scar is associated with increased infant proinflammatory immune responses
Source: Vaccine. 2017 Jan 5;35(2):273–82. doi: 10.1016/j.vaccine.2016.11.079 (PMC5357573; doi:10.1016/j.vaccine.2016.11.079)
Supplement: Supplementary Table 2 — Cytokine and chemokine responses in mothers with and without a BCG scar, and in their infants, measured by Luminex® assay. The values are shown as medians in pg/ml with the interquartile range in brackets. [file mmc2.docx]

**Supplementary Table 2**

| Cytokine/  Chemokine | Mothers without a BCG scar  (n=10) | Mothers with a BCG  scar  (n=16) | *p* value | Cytokine/Chemokine | Neonates of mothers  without a BCG scar  (n=10) | Neonates of mothers with a BCG scar  (n=16) | *p* value |
| --- | --- | --- | --- | --- | --- | --- | --- |
| IFN-γ | 24 (19, 36) | 29 (25, 36) | 0.215 | IFN-γ | 0 (0, 5) | 6 (2, 11) | **0.035** |
| TNF-α | 197 (60, 391) | 328 (107, 486) | 0.327 | TNF-α | 55 (29, 166) | 164 (69, 232) | 0.076 |
| IL-2 | 2 (2, 2) | 2 (2, 3) | 0.215 | IL-2 | 1.2 (1, 2) | 2 (1, 3) | 0.146 |
| IL-12p70 | 2 (1, 3) | 1 (1, 2) | 0.333 | IL-12p70 | 0 (0, 1) | 3 (1, 5) | **0.017** |
| IL-1β | 497 (62, 556) | 309 (85, 590) | 0.989 | IL-1β | 110.04 (52, 505) | 512 (82, 557) | 0.076 |
| IL-6 | 10792 (5181, 10843) | 10814 (6235, 10877) | 0.504 | IL-6 | 10069 (7045, 10795) | 9694 (5730, 12844) | 0.643 |
| IL-4 | 0.00 (0, 0) | 0.147 (0, 2) | **0.012** | IL-4 | 1.23 (1, 2) | 1 (1, 2) | 0.070 |
| IL-13 | 1 (0, 2) | 1 (1, 3) | 0.219 | IL-13 | 2 (0.25, 2) | 2 (0, 7) | 0.443 |
| IL-10 | 105 (58, 160) | 135 (113, 197) | 0.085 | IL-10 | 77 (47, 119) | 132 (89, 198) | **0.010** |
| IL-17A | 19 (14, 24) | 19 (12, 26) | 0.89 | IL-17A | 19 (16, 23) | 24 (20, 27) | 0.085 |
| IP-10 | 3950 (866, 4028) | 3863 (2007, 3998) | 0.89 | IP-10 | 2085 (35, 3842) | 3847 (1796, 3854) | 0.076 |
| IL-8 | 11587 (9634, 12750) | 13423 (11466, 9634) | 0.256 | IL-8 | 15289 (8122, 17340) | 15499 (12230, 22671) | 0.215 |
| GM-CSF | 13 (10, 16) | 15 (12, 18) | 0.328 | GM-CSF | 46 (40, 54) | 56 (46, 63) | **0.046** |
| VEGF | 16 (7, 22) | 6 (2, 11) | **0.031** | VEGF | 8 (2, 15) | 55 (8, 75) | **0.035** |
| MCP-1 | 1399 (1092, 2407) | 1007 (118, 2110) | 0.391 | MCP-1 | 1166 (1084, 1480) | 1359 (1247, 1541) | 0.196 |
| MIP-1α | 486 (478, 597) | 506 (482, 601) | 0.328 | MIP-1α | 480.10 (478, 533) | 503 (449, 677) | 0.382 |
| MIP-1β | 1260 (0, 1437) | 587 (0, 1381) | 0.521 | MIP-1β | 1317 (850, 1381) | 1304 (0, 1377) | 0.683 |
