# Supplementary material for: Maternal BCG scar is associated with increased infant proinflammatory immune responses
Source: Vaccine. 2017 Jan 5;35(2):273–82. doi: 10.1016/j.vaccine.2016.11.079 (PMC5357573; doi:10.1016/j.vaccine.2016.11.079)
Supplement: Supplementary Table 3 — The association between maternal BCG scar and infant innate responses. The values are shown as crude and adjusted geometric means ratios (GMR) with 95% confidence interval (CI). [file mmc3.docx]

**Supplementary Table 3.**

| Chemokines/  Cytokines | Crude GMR,  95% CI | Adjusted GMR,  (95% CI) ^a^ |
| --- | --- | --- |
| IFN-γ | **2.69 (1.24, 5.83)** | **2.69 (1.15, 6.17)** |
| TNF-α | 2.04 (0.98, 4.36) | 1.99 (0.69, 5.89) |
| IL-2 | 1.25 (1.00, 1.57) | 1.23 (0.89, 1.70) |
| IL-12p70 | **2.06 (1.24, 3.43)** | **1.95 (1.10, 3.55)** |
| IL-1β | 1.90 (0.71, 5.25) | 1.55 (0.37, 6.61) |
| IL-6 | 0.98 (0.62, 1.55) | 0.89 (0.45, 1.74) |
| IL-4 | 1.23 (1.00, 1.51) | 1.20 (0.89, 1.62) |
| IL-13 | 1.35 (0.79, 2.34) | 1.17 (0.63, 2.24) |
| IL-10 | **1.82 (1.14, 2.88)** | **1.82 (1.07, 3.09)** |
| IL-17A | 1.12 (0.81, 1.55) | 1.10 (0.71, 1.70) |
| IP-10 | 5.01 (0.79, 30.90) | **6.76 (1.17, 38.02)** |
| IL-8 | 1.29 (0.91, 1.82) | 1.35 (0.78, 2.29) |
| GM-CSF | **1.19 (1.02, 1.39)** | 1.15 (0.95, 1.38) |
| VEGF | **3.97 (1.38, 11.40)** | **3.55 (1.07, 11.48)** |
| MCP-1 | 0.46 (0.14, 1.44) | 0.49 (0.10, 2.34) |
| MIP-1α | 0.76 (0.35, 1.62) | 0.68 (0.22, 2.09) |
| MIP-1β | 0.29 (0.02, 3.80) | 0.69 (0.05, 9.77) |

^a^ adjusted for maternal LTBI
