# Supplementary material for: Maternal BCG scar is associated with increased infant proinflammatory immune responses
Source: Vaccine. 2017 Jan 5;35(2):273–82. doi: 10.1016/j.vaccine.2016.11.079 (PMC5357573; doi:10.1016/j.vaccine.2016.11.079)
Supplement: Supplementary Table 4 — Pairwise spearman rank correlation coefficients for cytokines and chemokines. Correlation between the cytokines and chemokines was assessed using the spearman rho test. P values less than 0.05 are shown in bold with † sign. [file mmc4.docx]

**Supplementary table 4.**

|  | IFN-γ | TNF-α | IL-2 | IL-12 | IL-1β | IL-6 | IL-4 | IL-13 | IL-10 | IL-17A | IP-10 | IL-8 | GM-CSF | VEGF | MCP-1 | MIP-1α | MIP-1β |
| --- | --- | --- | --- | --- | --- | --- | --- | --- | --- | --- | --- | --- | --- | --- | --- | --- | --- |
| TNF-α | **0.75^†^** |  |  |  |  |  |  |  |  |  |  |  |  |  |  |  |  |
| IL-2 | **0.68^†^** | **0.62^†^** |  |  |  |  |  |  |  |  |  |  |  |  |  |  |  |
| IL-12 | **0.42^†^** | 0.17 | **0.42^†^** |  |  |  |  |  |  |  |  |  |  |  |  |  |  |
| IL-1β | **0.51^†^** | **0.58^†^** | **0.76^†^** | **0.45^†^** |  |  |  |  |  |  |  |  |  |  |  |  |  |
| IL-6 | 0.22 | 0.23 | **0.61^†^** | 0.26 | **0.63^†^** |  |  |  |  |  |  |  |  |  |  |  |  |
| IL-4 | **0.69^†^** | **0.64^†^** | **0.98^†^** | **0.43^†^** | **0.81^†^** | **0.57^†^** |  |  |  |  |  |  |  |  |  |  |  |
| IL-13 | **0.50^†^** | 0.23 | **0.60^†^** | 0.23 | **0.39^†^** | 0.25 | **0.58^†^** |  |  |  |  |  |  |  |  |  |  |
| IL-10 | **0.46^†^** | **0.61^†^** | **0.56^†^** | 0.26 | **0.68^†^** | **0.40^†^** | **0.62^†^** | 0.19 |  |  |  |  |  |  |  |  |  |
| IL-17A | **0.40^†^** | **0.40^†^** | **0.48^†^** | 0.29 | 0.25 | 0.16 | **0.48^†^** | 0.06 | 0.40 |  |  |  |  |  |  |  |  |
| IP-10 | -0.01 | -0.14 | -0.05 | -0.08 | 0.10 | 0.21 | -0.01 | -0.10 | 0.07 | -0.07 |  |  |  |  |  |  |  |
| IL-8 | **0.71^†^** | **0.74^†^** | **0.76^†^** | **0.41^†^** | **0.59^†^** | **0.37^†^** | **0.73^†^** | 0.33 | **0.53^†^** | **0.45^†^** | -0.23 |  |  |  |  |  |  |
| GM-CSF | **0.54^†^** | 0.20 | **0.50^†^** | **0.60^†^** | **0.48^†^** | 0.21 | **0.47^†^** | **0.50^†^** | 0.30 | 0.10 | 0.16 | 0.35 |  |  |  |  |  |
| VEGF | **0.42^†^** | 0.26 | **0.43^†^** | **0.93^†^** | **0.53^†^** | 0.27 | **0.46^†^** | 0.30 | 0.35 | 0.28 | -0.07 | **0.42^†^** | **0.56^†^** |  |  |  |  |
| MCP-1 | **0.48^†^** | 0.35 | 0.27 | -0.10 | 0.17 | -0.01 | 0.29 | 0.12 | 0.27 | **0.45^†^** | 0.15 | **0.39^†^** | 0.11 | -0.09 |  |  |  |
| MIP-1α | **0.37^†^** | 0.50 | **0.67^†^** | 0.25 | **0.62^†^** | **0.44^†^** | **0.73^†^** | **0.45^†^** | **0.59^†^** | 0.33 | -0.12 | **0.51^†^** | 0.32 | 0.30 | 0.35 |  |  |
| MIP-1β | 0.04 | -0.03 | 0.03 | 0.06 | -0.14 | -0.10 | -0.01 | -0.05 | 0.08 | **0.44^†^** | -0.18 | 0.01 | -0.01 | 0.10 | 0.16 | -0.14 |  |
| RANTES | -0.09 | -0.04 | -0.17 | -0.09 | 0.05 | 0.05 | -0.01 | -0.01 | 0.07 | **-0.48^†^** | 0.21 | **-0.41^†^** | 0.03 | 0.01 | -0.17 | -0.02 | -0.16 |

† *p*<0.05. Statistically significant correlations are shown in bold.
