# Supplementary material for: Maternal BCG scar is associated with increased infant proinflammatory immune responses
Source: Vaccine. 2017 Jan 5;35(2):273–82. doi: 10.1016/j.vaccine.2016.11.079 (PMC5357573; doi:10.1016/j.vaccine.2016.11.079)
Supplement: Supplementary Fig. 1 — Cytokine and chemokine responses to innate stimuli, showing responses in maternal blood post-delivery (A) and cord blood (B). Clear and grey bars represent infants of mothers without and with LTBI, respectively. The horizontal lines represent the median. Statistically significant differences are shown by asterisk (∗). [file mmc5.docx]

**PAM3Cys-Ser (TLR 1/2 agonist) FSL-1 (TLR 2/6 agonist)**


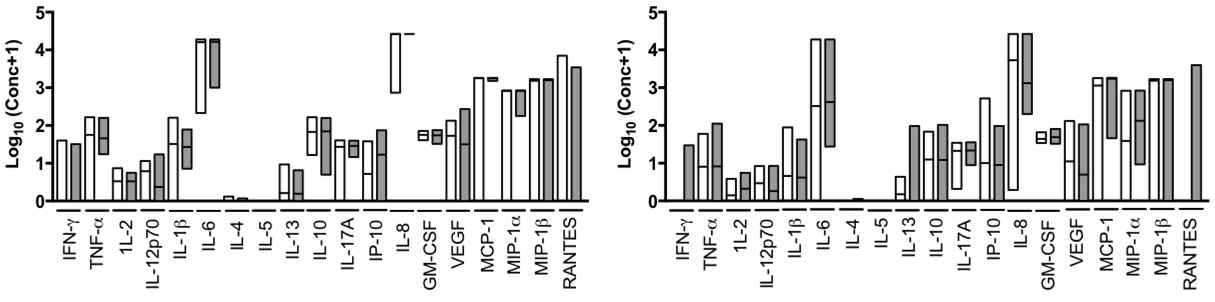


**LPS (TLR 4 agonist) CL097 (TLR 7/8 agonist**)


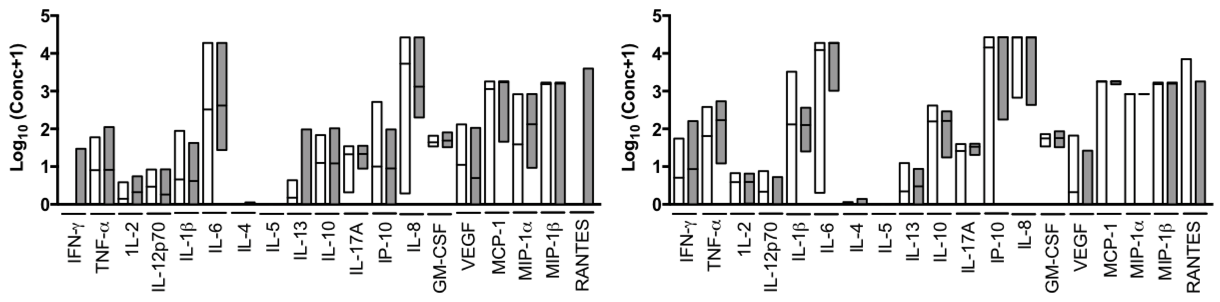


**Supplementary Figure 1A**.

**CpGODN2006 (TLR 9 agonist) Mannan (DC-SIGN agonist)**


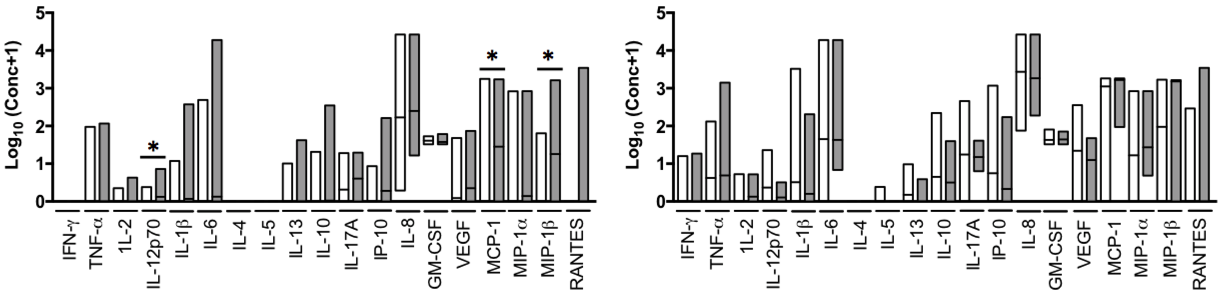


**Curdlan (Dectin-1 agonist)**


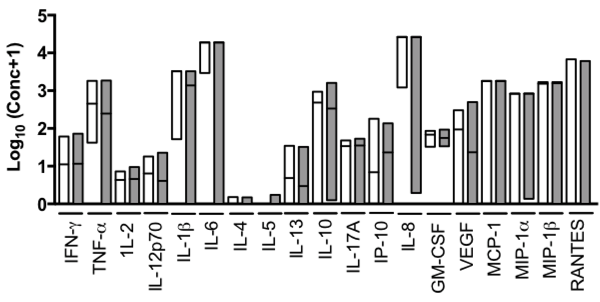


**Supplementary Figure 1B.**
