# Supplementary material for: Maternal BCG scar is associated with increased infant proinflammatory immune responses
Source: Vaccine. 2017 Jan 5;35(2):273–82. doi: 10.1016/j.vaccine.2016.11.079 (PMC5357573; doi:10.1016/j.vaccine.2016.11.079)
Supplement: Supplementary Fig. 2 — Cytokine and chemokine responses to innate stimuli, showing responses in maternal blood post-delivery (A) and cord blood (B). Clear and grey bars represent infants of mothers without and with a BCG scar, respectively. The horizontal lines represent the median. Statistically significant differences are shown by asterisk (∗). [file mmc6.docx]

**PAM3Cys-Ser (TLR 1/2 agonist) FSL-1 (TLR 2/6 agonist)**


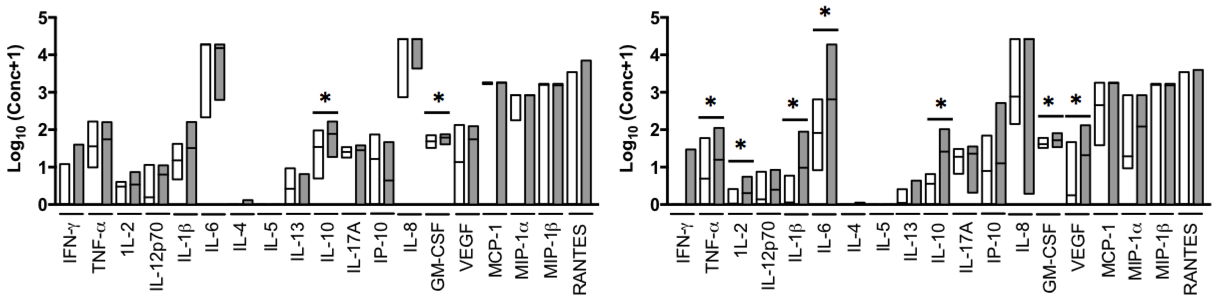


**LPS (TLR 4 agonist) CL097 (TLR 7/8 agonist**)


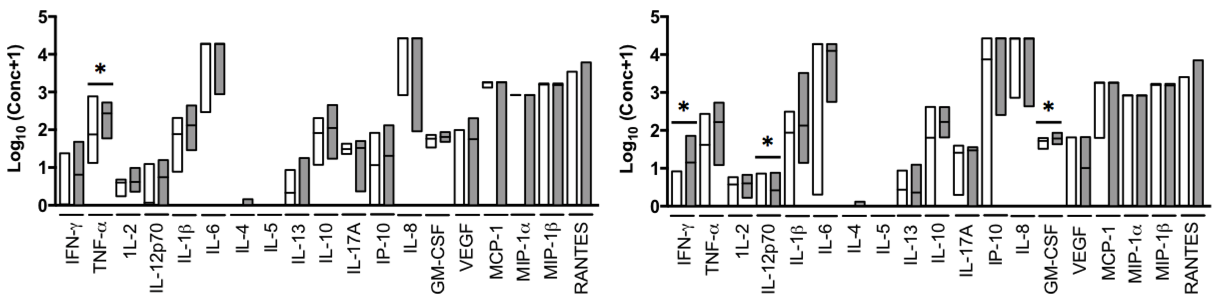


**Supplementary Figure 2A.**

**CpGODN2006 (TLR 9 agonist) Mannan (DC-SIGN agonist)**


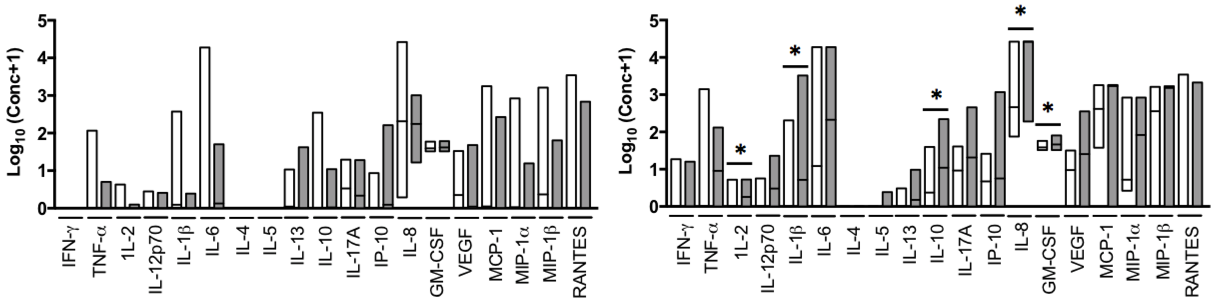


**Curdlan (Dectin-1 agonist)**


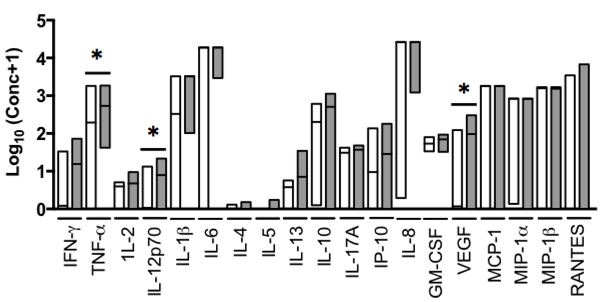


**Supplementary Figure 2B.**
