# Supplementary figures and images for: Maternal BCG scar is associated with increased infant proinflammatory immune responses
Source: Vaccine. 2017 Jan 5;35(2):273–82. doi: 10.1016/j.vaccine.2016.11.079 (PMC5357573; doi:10.1016/j.vaccine.2016.11.079)

**A**


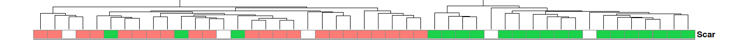

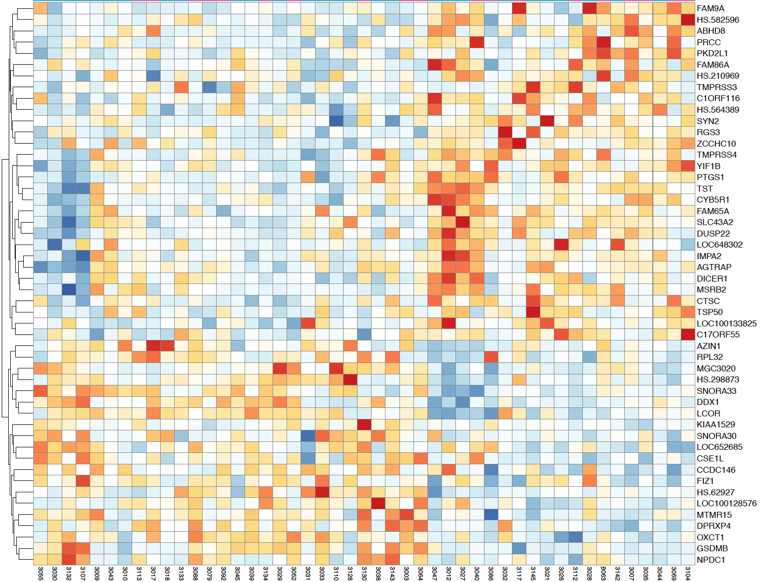

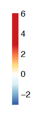

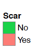


**B**


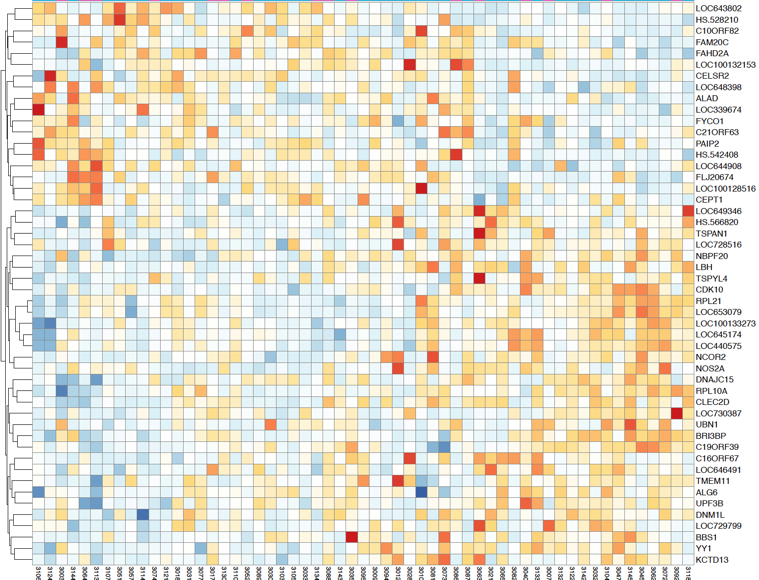

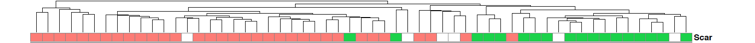

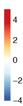

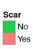


**Supplementary Figure 3.**

Supplement: Supplementary Fig. 3 — Heatmap showing the level of expression of the differentially expressed genes between infants of mothers with and without a BCG scar measured at one (A) and six (B) weeks post-BCG immunisation. The genes selected are the top significantly differentially expressed genes selected on fold change (1.3 fold up- or down- regulation) and p value (p < 0.05) basis. The colour scale shows the level of gene expression (scaled across infants) using z score, where red and green correspond to up- and down-regulation, respectively. [file mmc7.docx]
